# Antimicrobial activity of lignin from grape stalks: investigating the effect of different formulations

**DOI:** 10.1007/s11274-026-05158-8

**Published:** 2026-07-22

**Authors:** Ana C. Cassoni, Sara A. Cunha, Marta Vasconcelos, Manuela Pintado

**Affiliations:** Universidade Católica Portuguesa, CBQF - Centro de Biotecnologia e Química Fina – Laboratório Associado, Escola Superior de Biotecnologia, Rua Diogo Botelho 1327, Porto, 4169-005 Portugal

**Keywords:** Lignin, Antimicrobial, Agro-food residues, Foodborne pathogens

## Abstract

Lignin, a major component of lignocellulosic biomass, has gained attention as a potential antimicrobial agent. However, lignin's limited solubility often requires the use of harsh or non-eco-friendly solvents, which can hinder its applications and pose environmental concerns. This work aimed to evaluate the antimicrobial capacity of two lignin samples extracted from grape stalks using different methods: alkaline extraction and deep eutectic solvents. The antimicrobial activity of lignin was evaluated in three formulations: micronized, dissolved, and nanoparticles, against *Escherichia coli*, *Pseudomonas aeruginosa*, and *Staphylococcus aureus*. Results showed that dissolved lignin exhibited higher antimicrobial activity with expressive log reductions and total inhibition of bacterial growth at low concentrations (0.026-0.228 mg/mL). Micronized lignin demonstrated antimicrobial properties against *S. aureus* but only at higher concentrations (10-30 mg/mL). Lignin nanoparticles showed antimicrobial activity against *S. aureus* at much lower concentrations (0.250-0.468 mg/mL) than micronized lignin assays. Flow cytometry analysis revealed that dissolved lignin caused extensive damage to the bacterial cell membrane, leading to cell death. While dissolved lignin demonstrated the highest antimicrobial activity, micronized lignin and lignin nanoparticles also showed potential for specific applications. The development of sustainable extraction methods and green solvents is crucial for the successful utilization of lignin as an antimicrobial agent. This study highlights the potential of lignin from grape stalks as a natural antimicrobial alternative and discusses the importance of lignin's physical form in optimizing its antimicrobial performance and applications.

## Introduction

Lignin, a complex aromatic biopolymer abundant in lignocellulosic biomass, has gained significant attention due to its potential as a sustainable and renewable source of valuable compounds (Bajwa et al. 2019; Yoo et al. 2020). Lignin’s antimicrobial activity is a promising property, mainly for the development of natural and eco-friendly alternatives to synthetic antimicrobial agents (Chen et al. 2023; Das et al. 2024; Ndaba et al. 2020).

The antimicrobial properties of lignin have been attributed to its chemical structure, which includes phenolic hydroxyl, methoxyl, and carboxylic acid groups (Das et al. 2024). These functional groups interact with bacterial cell membranes, disrupting their integrity and leading to cell death (Chen et al. 2023; Gao et al. 2023). However, the application of lignin as an antimicrobial agent can been hindered by its limited solubility in water and most common organic solvents (Melro et al. 2018). To overcome this challenge, various strategies have been employed to modify lignin’s structure and enhance its solubility (de Oliveira et al. 2020; Figueiredo et al. 2018; Konduri and Fatehi 2015; Musilová et al. 2018; Sa’don et al. 2017). By improving lignin’s solubility, these methods also have potential to enhance its antimicrobial efficiency by increasing bioavailability and interaction with bacterial cells (Das et al. 2024; Wang et al. 2018).

Moreover, nanotechnology is also an interesting way to the development of lignin-based antimicrobial agents. Lignin nanoparticles (LigNP) have shown to be promising as effective antimicrobial agents due to their high surface area, improved dispersibility, and enhanced interactions with bacterial cell membranes (Ali et al. 2022; Figueiredo et al. 2018; Gao et al. 2023).

Despite the progress made in studying lignin’s antimicrobial properties, understanding and comparing the antimicrobial efficiency of lignin in different forms is still relevant. Hence, this study aims to compare the antimicrobial activity of lignin in three different formulations: micronized, dissolved, and nanoparticles. By evaluating the antimicrobial activity of alkaline lignin (AkL) and deep eutectic solvent lignin (DESL) against three bacterial strains (*Escherichia coli*, *Pseudomonas aeruginosa*, and *Staphylococcus aureus*), we aim to provide valuable insights into the relationship between lignin’s form, concentration, and antimicrobial efficiency.

This research demonstrates the significance of sustainable lignin extraction from agro-food residues and its evaluation in different formulations, contributing to the development of eco-friendly, versatile and bio-based antimicrobial agents based on circular economy principles.

## Materials and methods

Grape stalks from Vinhão variety were kindly provided by Quinta de Mascate (Vila Verde, Portugal). Biomass was dried, milled and stored at room temperature during the experiments.

High purity reagents used in this work (sodium hydroxide (NaOH), sulphuric acid (H_2_SO_4_), hydrochloric acid (HCl), lactic acid (LA), choline chloride (CC), Tween 80) were purchased from Merck KGaA (Darmstadt, Germany). Culture media Muller Hinton broth and agar were purchased from Biokar Diagnostics (Solabia Group, Paris, France). Peptone purchased from HiMedia Laboratories (Einhausen, Germany). Lignosulfonates were kindly provided by Altri SGPS S.A. (Porto, Portugal).

### Lignin extraction and micronization

Lignin was extracted from grape stalks using two previously optimized methods, alkaline and DES, as described by Cassoni et al. (2022). Briefly, the alkaline extraction method was carried out with 6% NaOH solution at 80 °C for 1 h, while the DES extraction utilized a mixture of lactic acid and choline chloride (5:1 molar ratio) at 120 °C for 5 h. Following extraction, the lignin extracts were dried and subjected to micronization using Planetary Ball Mill PM 100 (Retsch GmbH, Germany). The alkaline lignin (AkL) was milled for 5 min, and the DES lignin (DESL) for 20 min until a homogenous fine powder was obtained.

### Lignin solubilization

Micronized lignin samples were solubilized at a concentration of 2 mg/mL in Tween 80 (10%, w/w) and subjected to constant agitation at 100 rpm for 24 h to ensure maximum solubilization. Subsequently, samples were centrifuged to separate undissolved lignin and the supernatant was analyzed spectrophotometrically using a Shimadzu UV-Vis spectrophotometer (mini-1240, Kyoto, Japan) at a wavelength of 280 nm (Ajao et al. 2018). Solubilized lignin concentration was determined by interpolating the measured absorbance values using a previously established calibration curve (Cassoni et al. 2025).

### Lignin nanoparticles

Lignin nanoparticles were produced by ultrasonication as described by Cassoni at al. (2025). Briefly, micronized lignin samples were dispersed in a Tween 80 solution (0.05% w/w) and submitted to ultrasonication using the Ultrasonic processor Q700X (Qsonica, Newtown, CT, USA) with a 10 mm tip. The samples were maintained in an ice bath, under constant agitation to avoid samples overheating. After ultrasonication, nanoparticle solutions were concentrated 2-fold through evaporation in an oven at 45 °C. The effect of the evaporation step on nanoparticle stability was previously assessed (Cassoni et al. 2025), confirming no aggregation for DESL nanoparticles and negligible aggregation for AkL nanoparticles.

### Antimicrobial assays

Antimicrobial activity of the lignin samples was evaluated against three bacterial strains: *Escherichia coli* (ATCC 25922), *Pseudomonas aeruginosa* (ATCC 27853) and *Staphylococcus aureus* (ATCC 25923). The bacterial strains were maintained on Mueller Hinton agar plates and subcultured in Mueller Hinton broth for 24 h at 37 °C prior to the assay. Following incubation, the bacterial suspensions were adjusted to an optical density at 600 nm (OD600) between 0.08 and 0.13, corresponding to approximately 10^8^ colony-forming units per milliliter (CFU/mL). The suspensions were then diluted to a final concentration of 10^6^ CFU/mL for use in the antimicrobial assay.

The antimicrobial assay was performed in 24-well plates. Each well contained varying concentrations of the lignin extracts (Table 1) and the prepared bacterial inoculum, with a final volume of 1 mL per well. Lignosulfonates, a commercially available water-soluble lignin produced as a byproduct of the sulfite pulping process, were also included as an industry benchmark, at a concentration of 5 mg/mL.

**Table 1:** Lignin samples concentration for each antimicrobial assay (micronized lignin, dissolved lignin and lignin nanoparticles)

Concentration (mg/mL)	AkL		DESL	
	Initial	Final	Initial	Final
**Micronized Lignin**	1, 2, 5, 10, 20 and 30			
**Dissolved Lignin**	1.14 (± 0.01)	0.228 to 0.057	0.52 (± 0.01)	0.104 to 0.026
**Lignin Nanoparticles**	0.94 (± 0.05)	0.468	0.50 (± 0.04)	0.250

For micronized lignin, masses of 1, 2, 5, 10, 20, and 30 mg were directly added to the wells, followed by 1 mL of bacterial inoculum, resulting in a solid-state dispersion. The dissolved lignin samples were diluted 5-fold in the wells, in order to have a maximum concentration of Tween 80 of 2% (w/w). Hence, the final concentrations of the dissolved lignin in the wells ranged from 0.228 to 0.057 mg/mL for AkL and 0.104 to 0.026 mg/mL for DESL, as half and quarter concentrations were also tested. For the lignin nanoparticles, 0.5 mL of the nanoparticle suspension was combined with 0.5 mL of bacterial inoculum in each well, resulting in final concentrations of 0.468 mg/mL for AkL and 0.250 mg/mL for DESL. Positive controls were tested according to the volume of bacteria inoculum on each test. Negative controls consisting of Tween 80 (2% w/w) were also included in the assay.

The plates were incubated at 37 °C for 24 h – for micronized lignin samples, the plates were maintained under continuous agitation (40 rpm). Following incubation, 0.1 mL of the bacterial suspension from each well was transferred into 0.9 mL of sterile peptone water (1% w/v) and serial dilutions (from 10^− 1^ to 10^− 8^) were performed. From each dilution, 20 µL were plated onto Mueller-Hinton agar plates. The plates were incubated at 37 °C for 24 h to allow bacterial growth, and the number of colonies was counted to determine CFU/mL.

### Flow cytometry

Flow cytometry analysis was performed to assess the effect of lignin samples on bacterial cell viability and membrane integrity. The analysis was performed using a BD AccuriTM C6 Flow Cytometer (BD, New Jersey, USA) with the BDTM Cell Viability Kit, which uses a combination of thiazole orange (TO) and propidium iodide (PI) dyes. This dual-staining approach enables the rapid and straightforward differentiation of live, dead, and damaged bacterial cells. TO is a cell-permeant dye that stains all cells, enabling the discrimination of cells from background electronic noise or debris. PI is a membrane-impermeant dye that only enters cells with compromised membranes, indicative of dead or damaged cells. Therefore, the simultaneous use of TO and PI allows the classification of bacterial populations into three categories: live, dead, and damaged cells.

For this analysis, dissolved lignin samples were selected due to the incompatibility of micronized lignin and LigNPs samples with the flow cytometer. The lignin concentrations exhibiting the strongest antimicrobial activity against each bacterial strain were chosen for evaluation.

Samples were prepared as described by Lopes et al. (2025). Following exposure to the lignin samples, bacterial cells were harvested by centrifugation at 5000 rpm for 10 min. The resulting pellet was washed with phosphate-buffered saline (PBS) and subjected to a second centrifugation step under the same conditions. The washed cells were then resuspended in 500 µL of PBS and stained with 5 µL each of TO and PI. The stained samples were incubated at room temperature in the dark for a minimum of 5 min before analysis on the flow cytometer. Data acquisition and analysis were performed using a dot plot with FL1 and FL3 detectors to detect TO and PI, respectively. Fluorescence signals from TO and PI staining were collected as logarithmic signals. A gate in the Forward scatter (FS) vs. Sideward scatter (SS) plot was preset to selectively include the bacterial population, excluding artifacts and ensuring accurate fluorescence analysis of the relevant cells (Manoil et al. 2016). The bacterial populations were gated and differentiated into live, damaged, and dead cells, following the manufacturer’s recommendations for the BDTM Cell Viability Kit and using the plots of positive (live cells) and negative (dead cells) controls.

## Results

Lignin extraction yielded 32% (± 6.2) with a purity of 78.7% (± 1.76) for AkL, while DESL exhibited better results with a higher yield of 50.2% (± 2.3) and purity of 89.7% (± 1.8). Upon micronization, volume-weighted mean particle sizes were ~ 79.7 μm for AkL and ~ 45.8 μm for DESL. Solubilization of the micronized lignins in Tween 80, resulted in maximum solubilized lignin concentrations of 1.14 (± 0.01) mg/mL for AkL and 0.52 (± 0.01) mg/mL for DESL, as determined by spectrophotometric analysis. Uniform, spherical lignin nanoparticles were successfully produced, with average sizes of 289.5 (± 0.69) nm for AkL and 234.6 (± 0.46) nm for DESL and zeta potentials of approximately − 30 mV. The polydispersity index (PDI) for both lignin nanoparticle samples was below 0.200, indicating a monodisperse distribution.

These results, which have been previously published in our earlier works (Cassoni et al. 2022, 2025), provide a base for the present study focusing on the antimicrobial properties of the extracted lignins. As such, the details of the extraction, solubilization, and nanoparticle production processes are presented here in summary to contextualize the antimicrobial assays that follow.

### Antimicrobial assays

#### Micronized lignin

The antimicrobial activity of solid-state micronized lignin samples (micro-AkL and micro-DESL) against the three tested strains was evaluated using a range of concentrations (1, 2, 5, 10, 20, and 30 mg/mL). Results are presented as log reduction (CFU/mL) compared to the positive control and varied depending on the lignin type, concentration, and bacterial strain (Table 2).

**Table 2:** Antimicrobial activity of micronized lignin samples (alkaline and deep eutectic solvents) against three bacterial strains. n.d. – non defined; TI – total inhibition

Sample	Concentration (mg/mL)	Log reduction (CFU/mL)		
		*E.coli*	*P. aeruginosa*	*S. aureus*
AkL	30	1.87 (± 0.12)	1.96 (± 0.02)	TI
	20	1.08 (± 0.02)	1.75 (± 0.05)	TI
	10	0.32 (± 0.06)	1.27 (± 0.01)	TI
	5	n.d.	n.d.	TI
	2	n.d.	n.d.	2.33 (± 0.05)
	1	n.d.	n.d.	2.29 (± 0.31)
DESL	30	2,01 (± 0.07)	2.19 (± 0.11)	TI
	20	1.78 (± 0.01)	1.93 (± 0.04)	TI
	10	-0.02 (± 0.14)	0.60 (± 0.13)	4.19 (± 0.16)
	5	n.d.	n.d.	2.06 (± 0.24)
	2	n.d.	n.d.	1.30 (± 0.02)
	1	n.d.	n.d.	0.83 (± 0.03)

As expected, higher lignin concentrations were necessary to achieve substantial reductions in bacterial growth, particularly for *E. coli* and *P. aeruginosa*. Even high concentrations of micronized lignin (30 mg/mL) were ineffective in inhibiting bacterial growth. In contrast, low concentrations of micronized lignin (5 mg/mL) effectively inhibited the growth of *S. aureus*, which is the most susceptible strain.

Comparing the two lignins, the performance was very similar against *E.coli* and *P.aeurginosa*. However, micro-AkL performed better than micro-DESL against *S. aureus*. This difference may be due to variations in the chemical composition and structure of the lignins resulting from the different extraction methods employed (Alam et al. 2024; García et al. 2012 a; Ndaba et al. 2020).

#### Dissolved lignin

Like the micronized lignin tests, the results demonstrate the antimicrobial activity of dissolved lignins (AkL and DESL) in terms of log reduction (CFU/mL) relative to the positive control (Table 3). Due to the limitations of lignin dissolution, the concentrations tested were very low (from 0.228 to 0.025 mg/mL). However, results show that dissolved lignin exhibits notable antimicrobial activity.

**Table 3:** Antimicrobial activity of dissolved lignin samples (alkaline and deep eutectic solvents) against three bacterial strains. n.d. – non defined; TI – total inhibition

Sample	Concentration (mg/mL)	Log reduction (CFU/mL)		
		E.coli	*P*. aeruginosa	S. aureus
AkL	0.228	4.95 (± 0.07)	TI	TI
	0.114	1.40 (± 0.08)	TI	TI
	0.057	n.d.	3.71 (± 0.07)	4.89 (± 0.05)
DESL	0.104	6.18 (± 0.07)	TI	TI
	0.052	1.71 (± 0.13)	TI	TI
	0.026	n.d.	3.34 (± 0.12)	4.82 (± 0.01)
Lignosulfonates	5	0.81 (± 0.34)	1.94 (± 0.03)	TI

For *E. coli*, AkL (0.228 mg/mL) showed a log reduction of 4.95 ± 0.07, while DESL (0.104 mg/mL) achieved an even higher log reduction of 6.18 ± 0.07. These results indicate that both lignin samples exhibited strong antimicrobial activity against *E. coli*, with DESL being more effective at a lower concentration compared to AkL. As for *P. aeruginosa*, the antimicrobial activity was more evident, with total inhibition of bacterial growth observed for both AkL and DESL at the highest tested concentrations. Again, DESL was more effective with a log reduction of 3.34 ± 0.12 at a concentration of 0.026 mg/mL. Similarly, *S. aureus* exhibited high sensitivity to the dissolved lignin samples. Total inhibition was achieved by both lignin samples at the highest tested concentrations. At the lowest tested concentrations, log reductions of 4.89 ± 0.05 for AkL and 4.82 ± 0.01 for DESL were observed, respectively. This suggests that both *S. aureus* and *P. aeruginosa* are highly susceptible to the dissolved lignin samples, even at very low concentrations.

Regarding T80 (2%) control, it showed no antimicrobial activity against any of the tested strains, confirming that the inhibitory effects observed are attributable to the lignin fraction.

Comparing the antimicrobial activity of the two lignin samples, DESL appeared to be more effective than AkL against all three bacterial strains, since it achieved higher log reductions or total inhibition at lower concentrations. Lignosulfonates (5 mg/mL), used in this study to compare the performance of the AkL and DESL against a commercially available and well-characterized lignin, showed a lower antimicrobial activity against Gram-negative bacteria. For *E. coli* and *P. aeruginosa*, lignosulfonates achieved log reductions of only 0.81 ± 0.34 and 1.94 ± 0.03, respectively, in contrast to the higher activities demonstrated by AkL and DESL at lower concentrations (0.026–0.228 mg/mL). Interestingly, lignosulfonates showed complete inhibition of growth for *S. aureus* at the tested concentration. Overall, the lignin samples (AkL and DESL) showed higher antimicrobial activity compared to lignosulfonates, particularly against Gram-negative bacteria, highlighting their potential as alternative antimicrobial agents.

#### Lignin nanoparticles

Results presented in Table 4 demonstrate the antimicrobial activity of lignin nanoparticles (AkL and DESL) in terms of log reduction (CFU/mL) compared to the positive control.

**Table 4:** Antimicrobial activity of lignin nanoparticles (alkaline and deep eutectic solvents) against three bacterial strains

Sample	Concentration (mg/mL)	Log reduction (CFU/mL)		
		E.coli	*P*. aeruginosa	S. aureus
AkL	0.468	0.47 (± 0.02)	0.11 (± 0.03)	3.69 (± 0.21)
DESL	0.250	0.18 (± 0.03)	-0.14 (± 0.03)	1.57 (± 0.05)

Similarly to the dissolved lignin samples, the concentration of lignin nanoparticles was a limiting factor in this study, with the maximum concentrations achieved being relatively low (0.468 mg/mL for AkL and 0.250 mg/mL for DESL).

Results clearly indicate that both AkL and DESL nanoparticles had no antimicrobial effect against *E. coli* and *P. aeruginosa* at the tested concentrations. In contrast, lignin nanoparticles demonstrate antimicrobial effect against *S. aureus*. AkL nanoparticles achieved a log reduction of 3.69 (± 0.21), and DESL nanoparticles showed a log reduction of 1.57 (± 0.05). Comparing the antimicrobial activity of the two lignin nanoparticles, AkL nanoparticles appear to be more effective than DESL nanoparticles against *S. aureus*. However, it is important to note that the tested concentration of AkL nanoparticles was almost double that of DESL nanoparticles. Therefore, it is likely that with a higher concentration of DESL nanoparticles, antimicrobial activity would be similar to that of AkL nanoparticles.

### Flow cytometry

**Fig. 1:**
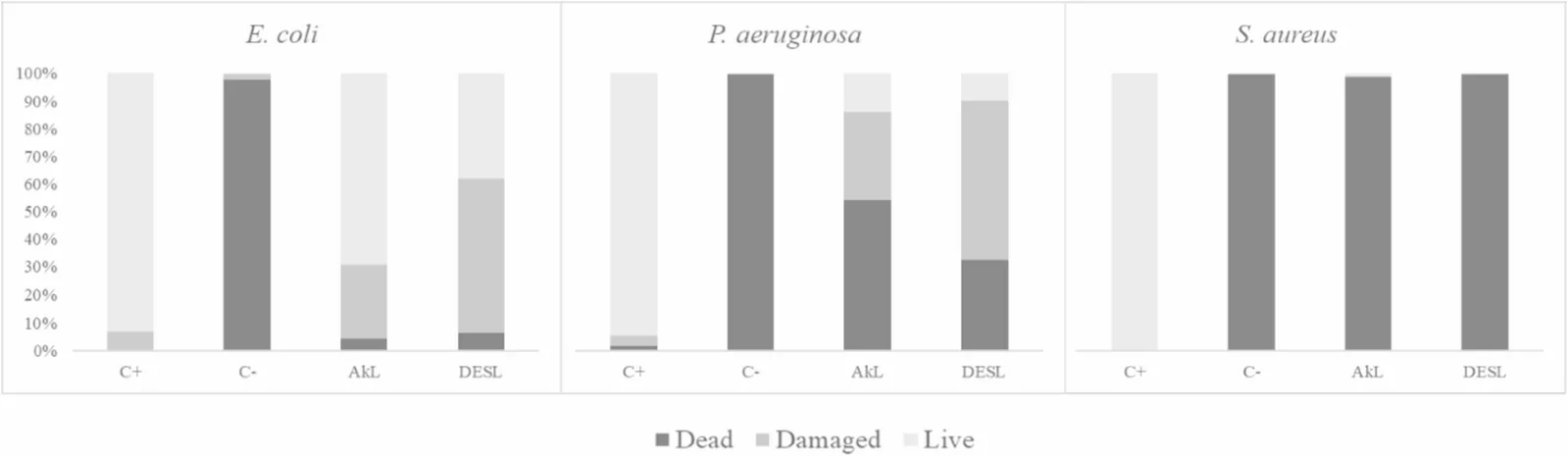
Flow cytometry of the dissolved lignin samples (alkaline - AkL and deep eutectic solvents - DESL) against three bacterial strains. Results expressed as live, damaged and dead bacterial cells. C+ - positive control; C- negative control

Flow cytometry results (Fig. 1) are consistent with the previously presented antimicrobial activity results.

For *E. coli*, lignin samples primarily caused cell damage, with a relatively low percentage of dead cells. The difference in the percentage of damaged cells between AkL and DESL treatments corroborates the antimicrobial activity results, where DESL achieved a higher log reduction compared to AkL. This suggests that DESL is more effective in compromising the cell membrane integrity of *E. coli*.

In the case of *P. aeruginosa*, the antimicrobial tests showed total inhibition of bacterial growth. The flow cytometry results reveal that the cells exposed to the lignin samples were both dead and damaged. AkL treatment resulted in a higher percentage of dead cells (41.5 ± 10.2%) compared to DESL (26.0 ± 8.4%). However, DESL treatment led to a higher percentage of damaged cells. The combined percentage of dead and damaged cells was similar for both lignin samples (approximately 70%). The presence of a small percentage of live cells, as detected by flow cytometry (a result not captured in the antimicrobial activity tests) can be attributed to the different detection thresholds of the two methods.

*S. aureus* exhibited the highest susceptibility to the dissolved lignin samples in the antimicrobial activity tests. The flow cytometry results corroborate these findings, showing that most *S. aureus* cells were dead when treated with the higher concentrations of both AkL and DESL. This clearly demonstrates the antimicrobial effect of the dissolved lignin samples on the bacterial strain.

Flow cytometry results provide insights into the mechanism of action of the lignin, demonstrating that the antimicrobial activity is, in part, due to cell membrane damage, ultimately leading to cell death. Hence, flow cytometry results complement the antimicrobial assay data and contribute to a more comprehensive understanding of the antimicrobial properties of the lignin extracts.

## Discussion

### Antimicrobial assays

#### Micronized lignin

The higher sensitivity of *S. aureus* to the micronized lignin samples may be attributed to lignin’s hydrophobic nature and its capacity to interact with bacterial membrane. Lignin establishes contact with bacterial cells through electrostatic attraction and hydrophobic interactions with membrane glycoproteins and lipid components (Raghuraman et al. 2005). Moreover, Gram-positive bacteria have a thicker peptidoglycan layer, which may facilitate the interaction and penetration of lignin compounds, such as phenolic compounds, thereby enhancing antimicrobial effects (Grossman et al. 2022; Verrillo et al. 2022). Additionally, Gram-negative bacteria have an outer membrane that contains lipopolysaccharides, which makes penetration of substances more difficult, thereby leading to greater resistance to antimicrobial agents (Impey et al. 2020).

Some studies have addressed lignin’s antimicrobial activity in its solid form. Dong et al. (2011) tested the antimicrobial activity of 100 mg/mL solid lignin from corn stover against several bacteria, including *S. aureus* and *E. coli*. Authors reported no antimicrobial activity against Gram-negative bacteria. A log reduction of 2.56 CFU/mL was achieved for *S. aureus*. In comparison, the micronized lignin from our study exhibits better antimicrobial activity against both Gram-positive and Gram-negative bacteria, requiring lower concentrations. Another study investigated the antimicrobial activity of a modified kraft lignin against *S. aureus* at 1 to 100 mg/mL achieving a maximum log reduction of 7.0 (Klein et al. 2019). Ebrahimi et al. (2025) also investigated lignin extracts from vine shoots against *E. coli* and *S. aureus*, at concentrations ranging from 0.4 to 25 mg/mL and obtained a minimum inhibitory concentration (MIC) of 3 mg/mL.

#### Dissolved lignin

Studies have investigated the antimicrobial properties of lignin, including lignin derived from agrofood residues. Zhang et al. (2025) investigated the antimicrobial activity of alkaline lignin extracted from rice straw (5 mg/mL) against *E. coli* and *S. aureus*. They reported a reduction of more than 50% in the growth of both bacterial strains and noted that ultrasound processing of rice straw enhanced the antimicrobial activity. Similarly, Kalinoski et al. (2020) demonstrated significant antimicrobial activity of lignin bio-oils extracted from corn stover, achieving inhibition of both Gram-positive and Gram-negative bacteria at a concentration of 3 mg/mL. Sunthornvarabhas et al. (2020) observed a higher susceptibility of Gram-positive bacteria to lignin extracted from sugarcane bagasse, with a MIC of approximately 8 mg/mL. Similar results were obtained using lignin extracted from sugarcane bagasse (El-Nemr et al. 2019). Using substantially higher concentrations of lignin (100 mg/mL) yielded a log reduction of 4.4 against *S. aureus* and no effect on *E. coli* (Alzagameem et al. 2019).

Overall, studies report efficient antimicrobial activities against both Gram-positive and Gram- negative bacteria. Notably, Gram-positive bacteria tend to be more susceptible to lignin, although this difference is less pronounced when micronized lignin is used. Comparing the concentrations of lignin that exhibited antimicrobial activity, the lignin extracted from grape stalks in this study demonstrated antimicrobial activity at lower concentrations (at least 10-fold lower) than those reported in the aforementioned studies.

Despite efforts to successfully extract lignin with antimicrobial properties from various agro-food residues, the antimicrobial activity of lignin remains relatively low compared to synthetic antimicrobial agents (Chen et al. 2023). This difference could be attributed to several factors, including the variability in lignin composition and structure depending on the source material and extraction method and the purity of the obtained lignin (Ndaba et al. 2020).

#### Lignin nanoparticles

The limited antimicrobial activity of lignin nanoparticles may be primarily due to the concentration tested; however, other factors should also be considered. The size and surface properties of the nanoparticles, as well as the specific interaction between the nanoparticles and the bacterial cell membrane, also play a crucial role. Additionally, residual Tween 80 adsorbed onto the nanoparticle surface during ultrasonication may shield active phenolic groups from direct contact with bacterial cells through steric hindrance (Li et al. 2016), reducing antimicrobial efficiency. Further optimization of nanoparticle formulation, including size reduction and surface functionalization, may enhance antimicrobial efficiency.

In a study conducted by Gao et al. (2023), lignin nanoparticles with a size range of 120–130 nm demonstrated effective inhibition of both *S. aureus* and *E. coli* growth at a concentration of 5 mg/mL. On the other hand, Tanganini et al. (2024) reported the inhibition of *S. aureus* growth using smaller lignin nanoparticles with a size of 80 nm at a remarkably low concentration of 0.025 mg/mL. However, these nanoparticles had no effect on Gram-negative bacteria. Freitas et al. (2020) tested the antimicrobial activity of lignin particles (from 100 to 300 nm) against *E. coli* at a concentration of 1 mg/mL. They observed that lignin particles reduced bacterial growth, with the intriguing finding that larger particles exhibited greater antimicrobial activity. The authors attributed this enhanced efficiency to the surface charge of the particles, which was closer to zero (-18.80 mV), suggesting that a more neutral surface charge may contribute to improved antimicrobial activity. In contrast, the lignin nanoparticles from the present study have a more negative surface charge of approximately − 30 mV, which could potentially hinder the interaction between nanoparticles and the bacterial cell surface, thereby reducing antimicrobial activity (Wang et al. 2017). Ali et al. (2022) investigated the antimicrobial activity of LigNPs with an average size of 197.5 nm and a highly negative surface charge of -41.7 mV. They reported MICs of 3.3 ± 0.96 mg/mL for *S. aureus* and 2.8 ± 0.89 mg/mL for *E. coli*. This indicates that the surface charge of lignin nanoparticles is not the sole factor influencing antimicrobial activity, and other factors, such as particle size, lignin source, and extraction method, may also play significant roles.

To gain a deeper understanding of the antimicrobial mechanism of lignin nanoparticles, additional studies investigating their interaction with bacterial cells would be valuable. Gao et al. (2023) performed a detailed study on how lignin nanoparticles act on bacteria. Scanning electron microscopy (SEM) revealed that the cell membranes of both bacteria were wrinkled and distorted after treatment with lignin nanoparticles, indicating compromised cell integrity. Additionally, alkaline phosphatase levels showed a significant increase, indicating damage to the cell wall. Cell membrane integrity was assessed by measuring changes in electrical conductivity and the absorbance of macromolecules. The conductivity of the culture medium increased after treating bacteria with lignin nanoparticles, suggesting altered cell membrane permeability. Transmission electron microscopy (TEM) revealed that the cytoplasmic content in bacteria decreased gradually after treatment with lignin nanoparticles. The cell wall structure of *S. aureus* was deformed, with marked incisions, while smaller lignin nanoparticles were found inside *E. coli*. The authors hypothesized that lignin nanoparticles not only destroy cells from the outside but may also be internalized into cells due to their small size, potentially affecting protein synthesis in ribosomes and disrupting normal bacterial metabolism.

#### Critical comparison of antimicrobial activity between the three lignin formulations

The antimicrobial activity of lignin was evaluated in three different formulations: micronized, dissolved, and nanoparticles. The results demonstrate that the formulation and concentration of lignin play a crucial role in its antimicrobial activity against the tested bacterial strains.

It should be noted that the concentrations tested for each formulation were substantially different (from 1 to 30 mg/mL for solid-state micronized lignin to 0.026–0.228 mg/mL for dissolved lignin and 0.250–0.468 mg/mL for nanoparticles), reflecting the limitations of each formulation. In this context, activity is discussed in terms of the minimum concentration at which a meaningful log reduction was observed for each formulation.

Overall, dissolved lignin demonstrated the highest antimicrobial activity at the lowest concentrations, followed by lignin nanoparticles and micronized lignin. This difference is particularly noticeable when comparing dissolved DESL (0.026 mg/mL) with micronized AkL (30 mg/mL), both achieving meaningful inhibition of *S. aureus*, but at concentrations approximately 1000-fold apart, a difference that reflects not only the physical state of lignin but also its distinct mode of interaction with bacteria. This trend could be partially attributed to the increased accessibility and interaction of lignin molecules with bacterial cells when in the dissolved state. The dissolution of lignin allows its molecules to be more readily available to interact with the bacterial cell surface, cell wall, and cell membrane, potentially leading to more efficient antimicrobial action (Das et al. 2024; Wang et al. 2018). This enhanced interaction may facilitate the proposed mechanisms of action of lignin, such as the induction of oxidative stress through the reactive oxygen species (ROS) mechanism and the penetration of low molecular weight lignin into the cell membrane (Chen et al. 2023; Kalinoski et al. 2020; Yang et al. 2018 b).

Micronized lignin particles interact with bacteria mainly through solid-state contact, with a more limited surface area available for interaction, which may result in lower antimicrobial efficiency. However, micronized lignin still holds value for potential applications. In this study, micronized lignin achieved total inhibition of *S. aureus* at concentrations above 5 mg/mL. The incorporation of micronized lignin into materials such as coatings and various polymer matrices, particularly in food packaging, could provide antimicrobial activity against foodborne bacteria (El-Nemr et al. 2019; Rai et al. 2017). Moreover, micronized lignin requires less processing than the other formulations of lignin tested, making it simpler and more cost-effective.

Lignin nanoparticles, on the other hand, have a higher specific surface area compared to micronized lignin particles due to their smaller size. This increased surface area can facilitate better interaction with bacterial cells, leading to enhanced antimicrobial activity compared to micronized lignin (Feng et al. 2024; Gao et al. 2023). The enhanced antimicrobial efficiency of lignin nanoparticles at lower concentrations compared to micronized lignin is consistent with this principle, though the precise mechanisms, including the particle size, surface charge, and lignin bioavailability, require further investigation. The unique properties of lignin nanoparticles and the potential for surface modification make them promising candidates for specific applications, such as targeted nanocarriers (Aguiar et al. 2020; Jalali et al. 2020; Maruyama et al. 2020), or as antimicrobial additives in nanocomposite materials (Luo et al. 2020; Yang et al. 2018 b).

Interestingly, some studies reported an opposite trend, with lignin nanoparticles exhibiting greater antimicrobial efficiency than dissolved lignin (Ali et al. 2022; Gao et al. 2023). This may be due to the chemical and physical changes that lignin undergoes during processing (dissolution or production of nanoparticles), which could alter its mechanism of action (Figueiredo et al. 2018), making it challenging to predict the most efficient formulation of lignin. Furthermore, as previously discussed, the source and extraction method of lignin, prior to its processing, also influence its structure and, consequently, its antimicrobial activity (Ndaba et al. 2020). Thus, the vast heterogeneity of lignin extracts hampers the prediction and comparison of their antimicrobial properties.

Other limitations of this study should also be acknowledged. The concentrations of dissolved lignin and lignin nanoparticles tested were limited by the solubility and production yield, which prevented evaluation across a broader concentration range. For lignin nanoparticles, only a single concentration per sample was assessed, limiting the ability to establish dose-response relationships and determine MIC values. Flow cytometry analysis was restricted to dissolved lignin samples due to the incompatibility of micronized lignin and nanoparticle formulations with the flow cytometer, which impeded a direct comparison across all three formulations. Finally, the antimicrobial assays were conducted against only three bacterial strains under in vitro conditions and therefore, the results may not fully reflect the activity against a broader range of relevant microorganisms, nor the complexity of real-world applications.

## Conclusions

In conclusion, this study demonstrates the antimicrobial potential of lignin extracted from grape stalks using traditional alkaline extraction and the green alternative deep eutectic solvents. The evaluation of lignin’s efficiency in micronized, dissolved, and nanoparticle formulations highlight the importance of its physical state and concentration in determining the antimicrobial activity. Dissolved lignin exhibited the highest activity, followed by lignin nanoparticles and micronized lignin. DESL showed higher efficiency compared to AkL, highlighting the potential of green methods in extracting lignin with antimicrobial properties. Flow cytometry revealed that the antimicrobial mechanism of dissolved lignin involves damage to the bacterial cell membrane.

The extraction of lignin from grape stalks aligns with the principles of circular economy and sustainable resource utilization. The successful demonstration of lignin’s antimicrobial properties in different physical states opens a wide range of potential applications. This study provides a foundation for the development of sustainable, bio-based antimicrobial solutions while promoting the valorization of agro-food waste. By using lignin from grape stalks and exploring its antimicrobial potential in various formulations, this research contributes to the advancement of green chemistry and the transition toward a more sustainable future.

## Data Availability

No datasets were generated or analysed during the current study.
